# Alcohol in the Atmosphere

**Published:** 1881-06

**Authors:** 


### ALCOHOL IN THE ATMOSPHERE.

According to certain researches of Muntz, recently communicated at the March 7, session of the French Academy of Science (Bulletin Generale de Therapeutique Medicale et Chirurgicale, March 30, 1881), alcohol is formed in great abundance on the surface of the earth, in the soil and at the sea bottom, from the decomposition of organic matter, and that, obedient to the laws of vapor tension, it passes into the atmosphere. These facts, if proven, will tend to vitiate certain medico-legal investigations dependent on the recognition of the presence of alcohol.

